# Towards racemizable chiral organogelators

**DOI:** 10.3762/bjoc.6.107

**Published:** 2010-10-06

**Authors:** Jian Bin Lin, Debarshi Dasgupta, Seda Cantekin, Albertus P H J Schenning

**Affiliations:** 1 Functional Organic Materials and Devices and Laboratory of Macromolecular and Organic Chemistry, Eindhoven University of Technology, PO Box 513, 5600MB Eindhoven, The Netherlands

**Keywords:** chirality, organogels, racemization, self-assembly

## Abstract

A chiral organogelator has been synthesized that can be racemized and self-assembled in apolar solvents whilst at higher concentrations organogels are formed. Field emission scanning and transmission electron microscopy revealed the formation of bundle fibrils that are able to gelate the solvent. ^1^H NMR studies showed hydrogen-bond interactions between the peptide head groups of neighbouring organogelator molecules. The enantiomerically pure organogelator can be racemized by the base DBU (1,8-diazabicyclo[5.4.0]undec-7-ene) as was evident from chiral high-performance liquid chromatography analysis.

## Introduction

Gelation represents a macroscopic manifestation of self-assembled molecules. Impressive supramolecular architectures have been reported in which the self-assembled molecules immobilize solvent to produce a gel phase. Carefully designed self complementary building blocks with co-added organizational information can make these gels responsive. In recent years, much effort has been devoted to the design and characterization of chiral self-assembled fibrillar networks that form organogels [1–2]. In such systems the chirality within a molecular building block is transcribed to nano- or mesoscale fibrous assemblies. These chiral structures represent excellent models for studying the emergence of specific shapes at a macroscopic level through cooperative interactions between molecules. In addition, helical assemblies possess a potential for applications in advanced materials and constrained media for chiral synthesis and separation [3–5].

The development of systems where the chiral supramolecular assembly responds to specific triggers, should facilitate the design of smart functional materials in which subtle molecular-scale changes have an impact on the macroscopic behavior. Most of the earliest stimuli-responsive gels undergo a UV-induced transformation, which can be reversed by visible light. For example, Shinkai and coworkers demonstrated that *trans*-*cis* isomerization of gelator compounds by UV/visible light could induce a gel-sol transition [6]. Feringa and coworkers have reported a chiral gelator in which the supramolecular organization of the chiral assemblies can be switched using UV/visible light combined with heating and cooling [7]. Chiral gels that respond to other stimuli such as metal ions [8], guest molecules [9] and temperature [10] have also been reported.

**Scheme 1:**
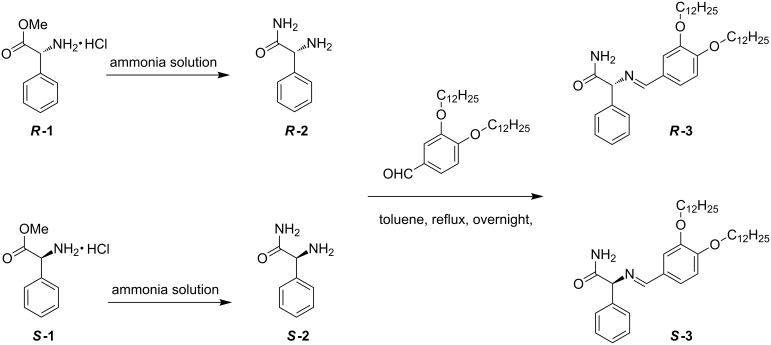
Synthesis of ***R***-**3** and ***S***-**3**.

In recent years, racemic gel fibers assembled from mixtures of enantiomeric building blocks have been described [11–14]. In most cases, the racemates were less efficient gelators than the pure enantiomers, and sometimes lead to crystallization [7]. Interestingly, Higashi and coworkers have observed that the separate enantiomers assemble into fibers with opposite helicity while the racemic mixture yield nanoscale, spherical structures [15]. In order to create chiral response materials based on such systems, it would be interesting to develop organogelators that can be racemized by a stimulus [16–17]. In the current manuscript we report our attempt to synthesize a racemizable chiral organogelator (Scheme 1). The molecular design of our organogelator (**3**) is based on a finding that the imine of 2-methylbenzaldehyde and phenylglycinamide can be racemized in the presence of the base DBU (1,8-diazabicyclo[5.4.0]undec-7-ene). Interestingly, a single solid chiral state from a nearly racemic mixture of this phenylglycinamide derivative was observed by so-called attrition-enhanced solid-phase enantioenrichment [18]. In order to obtain a molecule that is able to form a gel, 3,4-didodecyloxybenzaldehyde [19] was used in place of 2-methylbenzaldehyde.

## Results and Discussion

### Synthesis

**Figure 1:**
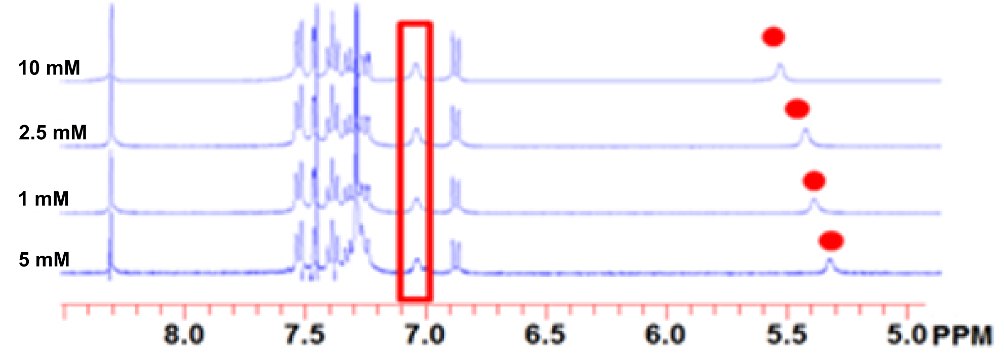
Concentration-dependent ^1^H NMR spectra of ***R***-**3** in chloroform (CDCl_3_). The red colours indicate the hydrogen resonances of the amide unit.

The synthesis of ***R***-**3** and ***S***-**3** is outlined in Scheme 1. First, concentrated aqueous ammonia was added to ester **1** to yield pure phenylglycinamide **2** [18]. Reaction of **2** with 3,4-didodecyloxybenzaldehyde [19] led to compound **3** in 50% yield (Scheme 1). Both ***R***-**3** and ***S***-**3** were purified by recrystallization and fully characterized. Chiral high-performance liquid chromatography (HPLC) showed an ee of more than 99% for the enantiomers. Interestingly, ^1^H NMR spectroscopy in chloroform revealed that the two amide protons of **3** behave differently at different concentration (Figure 1). The signal from one of the amide protons remains the same, even on the addition of a small amount DMSO-*d*_6_, indicating intramolecular hydrogen bonding. The other amide proton signal was downfield shifted upon increasing the concentration, in accord with intermolecular hydrogen bonding.

### Gel formation

**Figure 2:**
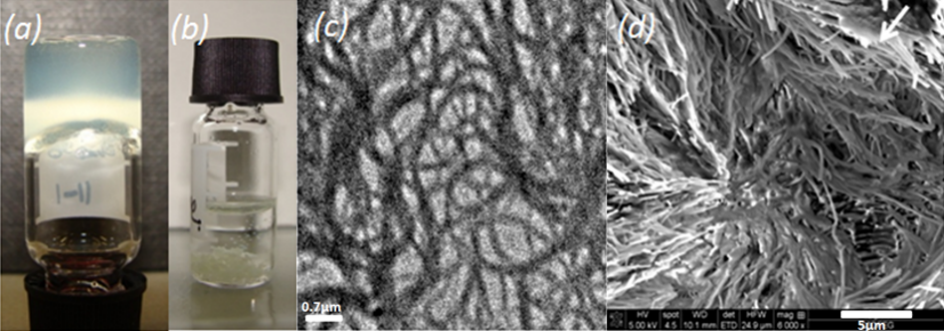
a) ***R***-**3** gel in octane (5 mM); b) octane solution containing a mixture of ***R***-**3** (2.5 mM) and ***S***-**3** (2.5 mM) after cooling from 80 °C to room temperature; c) TEM image of the xerogel of ***R***-**3** in octane; d) the surface morphology of the dried gel obtained from ***R***-**3** in octane (15 mM) observed by FESEM.

The gelation ability of ***R***-**3**, ***S***-**3** and racemic **3** was investigated using the “inverse flow” method. At room temperature, ***R***-**3** and ***S***-**3** were insoluble in octane. However, on heating at 80 °C, both ***R***-**3** and ***S***-**3** became soluble in octane and when cooled to room temperature, a stable self-supporting semi-transparent gel was formed (Figure 2a). For all the samples, the thermoreversible gelation process was followed at 0 °C, since the gelation time becomes much longer at lower concentrations at 20 °C. For ***R***-**3**, the critical gelation concentration (CGC) was detected by the failure of the whole mass to flow when the vial was turned upside down. The CGC value was 2.3 mM for ***R***-**3**, which means that ***R***-**3** can immobilize approximately 2700 molecules of octane per gelator molecule. The gel is thermoreversible, indicating that the first order phase transition is associated with gel melting and/or gel formation. When the racemate (***R***-**3** = ***S***-**3** = 2.5 mM) was heated and cooled to room temperature, precipitation was observed (Figure 2b).

The morphology of the organogel was further characterized by field emission scanning electron microscopy (FESEM) as well as with transmission electron microscopy (TEM). The TEM image (Figure 2c), of the dried ***R***-**3** gel in octane (1 mM) exhibited entangled fibrillar network formation although the concentration stays well below the CGC value (2.3 mM) in this solvent. The critical gelation concentration indicates the threshold at which, infinite percolation is achieved within a network system, although microgel network structures can still be observed below the CGC. The fibers have an average diameter is 60 ± 10 nm and lengths of tens of micrometers suggesting effective anisotropic growth. The morphology of the ***R***-**3** organogel in octane at a higher concentration (about 15 mM) also revealed a fibrillar structure, but in this case the fibrils were much bigger in size (nearly 150 nm in diameter) and in some parts they seemed to form two dimensional sheet like lamellar structures (FESEM image, Figure 2d).

**Figure 3:**
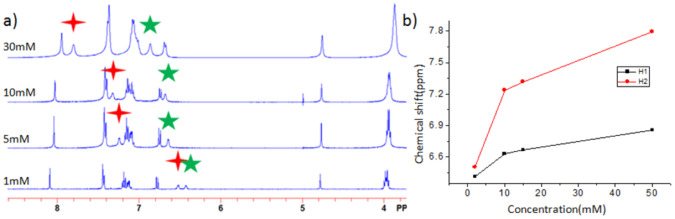
a) Concentration-dependent ^1^H NMR spectra of ***R***-**3** in cyclohexane-*d*_12_ and b) the shift of N–H signal of amide group versus concentration.

In order to investigate the molecular arrangement of the fibers in apolar solvent, variable concentration ^1^H NMR measurement were carried out in cyclohexane-*d*_12_. In contrast to the observations in chloroform, in cyclohexane solution upon increasing the concentration the observed chemical shifts of both the N–H protons were downfield shifted (Figure 3), consistent with an increased degree of intermolecular hydrogen bonding suggesting the formation of intermolecular hydrogen-bond interactions between the amide units. Most likely hydrogen bonded dimers or tetramers are formed in this apolar solvent. Our data suggest that these hydrogen-bonded dimers or tetramers subsequently stack via π-π interaction and via van der Waals interactions into bundled fibers.

### Racemization

**Figure 4:**
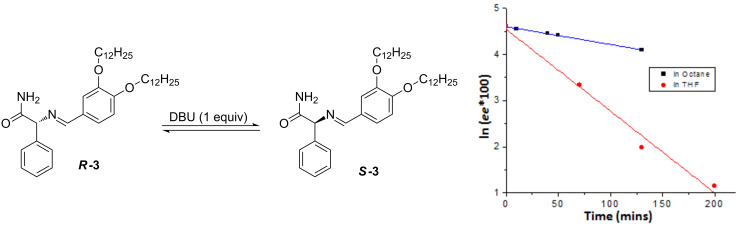
The evolution of ln(*ee* × 100) for the racemization of ***R***-**3** (1 mM) in the presence of 1 equivalent of DBU in octane and THF.

The racemization of ***R***-**3** was investigated by chiral HPLC. After adding 1 equivalent of DBU to a solution of ***R***-**3** in octane at room temperature, samples were collected over time and the enantiomeric purity was measured. Racemization was not observed in the gel state (5 mM) after 18 hours, whilst after heating and cooling precipitation was observed. At a concentration of 1 mM, where self-assembled fibers are present, the ee of the solution decreased in time. Figure 4 shows a plot of ln(*ee* × 100) versus time for the racemization of ***R***-**3** [18]. The first order kinetics expression: ln(*ee*(*t*)) = ln(*ee*(*0*)) + *kt* could be used to fit the experimental results and gave a t_1/2_ of about 177 min. Under the same conditions, the racemization rate in THF is much faster with a t_1/2_ of about 37 min. This difference between the two solvents is most likely due to the difference in polarity and the fact that ***R***-**3** is self-assembled in octane and molecularly dissolved in the more polar THF.

## Conclusion

This work demonstrates a route towards racemizable chiral organogelators. Remarkably, our pure enantiomeric gelator could not be racemized in the gel phase, while racemization takes place in the self-assembled fiber state. We are currently studying this class of organogelator for the creation of responsive gels and attrition-enhanced solid-phase enantioenrichment phenomena [20].

## Experimental

### General

All chemicals were purchased from Aldrich. Non-deuterated solvents were purchased from Biosolve. All other solvents and chemicals were used as received. Deuterated solvents were obtained from Cambridge Isotope Laboratories, United States. NMR spectra were recorded with a Varian Mercury NMR Spectrometer. IR spectra were measured on a Perkin Elmer 1600 FT-IR. MALDI-TOF MS spectra were measured on a Perseptive DE Voyager Mass Spectrometer with α-cyano-4-hydroxycinnamic acid as the matrix.

### Synthesis

Phenylglycinamide ***R***-**2**, ***S***-**2**: The ester salt ***R***-**1**, ***S***-**1** (5.04 g, 25 mmol) was stirred with concentrated ammonia solution (20 mL) at room temperature overnight. Subsequently, the product was precipitated, filtered, and washed with cold water. The resulting white solid was recrystallized from ethanol to afford 2.65 g of colourless crystals (71%) of ***R***-**2**, ***S***-**2**. ^1^H NMR (CDCl_3_, 400 MHz): 7.43 (m, 2H, ArH); 7.31 (m, 3H, ArH); 4.84 (br, 4H, NH_2_); 4.44 (s, 1H, COCH). MALDI-TOF MS (calc MW = 150.08, C_8_H_10_N_2_O): 150.97 [M + H]^+^. IR ν (cm^−1^): 3339; 3073; 1660; 1454; 1405; 1271; 869; 699.

***R***-**3**, ***S***-**3**: To a solution of 3,4-didodecyloxybenzaldehyde (237 mg, 0.5 mmol) in toluene, was added compound ***R***-**2**, ***S***-**2** (75 mg, 0.5 mmol) and the mixture was refluxed overnight. After the reaction was complete, the solvent was removed. The resulting yellow solid was washed with methanol and *n*-hexane to yield 150 mg ***R***-**3**, ***S***-**3** (50%). ^1^H NMR (CDCl_3_, 400 MHz): 8.19 (s, 1H, CH=N); 7.48 (m, 2H, ArH); 7.42 (d, *J* = 1.2 Hz, 1H, ArH); 7.34 (m, 2H, ArH); 7.30 (m, 1H, ArH); 7.23 (dd, *^1^J* = 6.3Hz, *^2^J* = 1.2Hz, 1H, ArH); 7.03 (d, *J* = 2.8Hz, 1H, CONH_2_); 6.89 (d, *J* =6.3Hz, 1H, ArH); 5.49 (d, *J* = 2.8 Hz, 1H, CONH_2_); 4.95 (s, 1H, COCH); 4.05 (m, 4H, OCH_2_, OCH_2_); 1.83 (m, 4H, CH_2_) ; 1.58 (m, 4H, CH_2_) ; 1.46 (m, 4H, CH_2_) ; 1.83 (m, 32H, CH_2_) ; 1.83 (m, 6H, CH_3_). ^13^C NMR (CDCl_3_, 100 MHz): 199.20; 174.34; 162.90; 152.33; 149.32; 144.98; 139.45; 128.65; 128.43; 127.83; 127.25; 123.52; 112.46; 111.78; 69.35; 69.07; 31.90; 29.62; 29.42; 29.34; 29.25; 29.08; 26.03; 25.96; 22.66; 14.09. MALDI-TOF MS (calc MW = 606.48, C_39_H_62_N_2_O_3_): 607.51 [M + H]^+^. IR ν (cm^−1^): 3403; 2918; 2849; 1652; 1514; 1263; 1120; 719. M.p. 80.5 °C. (*R*)-enantiomer: [α]^25^_D_ 4.8 (*c* 0.01, CHCl_3_), (*S*)-enantiomer: [α]^25^_D_ −4.4 (*c* 0.01, CHCl_3_).

ee Determination by HPLC [18]: The octane solution (2 mL) of the imine (1 mM) and DBU (1 equiv) was mixed with 1 mL of 0.25 M HCl solution and the aqueous solution extracted two times with 1 mL of CHCl_3_. The remaining aqueous solution of phenylglycine amide HCl salt was used as such for the ee determination by the following HPLC method. Column; crownether Cr (+) 150 x 4.6 mm ID, eluent; aqueous HClO_4_ pH = 1.2 / methanol 90/10 v/v %, flow: 1 mL/min, temperature, 25 °C, detection: λ = 220 nm, detection limit: 0.01 area %.

Electron Microscopy: The field emission scanning electron microscopy was performed on dried gel samples. The samples (15 mM in octane) were first coated with gold by the sputtering technique and then observed under a FEI Quanta 3D FEG microscope. The transmission electron microscopy was carried out by drop casting a solution (1 mM in octane) on a carbon coated copper grid and viewed under FEI Tecnai 20. TEM grids (R2/2 Quantifoil Jena) were purchased from Quantifoil.
